# High Intensity Concentric-Eccentric Exercise Under Hypoxia Changes the Blood Metabolome of Trained Athletes

**DOI:** 10.3389/fphys.2022.904618

**Published:** 2022-06-23

**Authors:** Tobias Dünnwald, Giuseppe Paglia, Günter Weiss, Vanna Denti, Martin Faulhaber, Wolfgang Schobersberger, Henning Wackerhage

**Affiliations:** ^1^ Institute for Sports Medicine, Alpine Medicine and Health Tourism (ISAG), UMIT TIROL, Private University for Health Sciences, Medical Informatics and Technology, Hall in Tirol, Austria; ^2^ School of Medicine and Surgery, University of Milano-Bicocca, Vedano al Lambro (MB), Italy; ^3^ Department of Internal Medicine II, Medical University of Innsbruck, Innsbruck, Austria; ^4^ Department of Sport Science, University of Innsbruck, Innsbruck, Austria; ^5^ Tirol-Kliniken GmbH, Innsbruck, Austria; ^6^ Department of Sport and Health Sciences, Technische Universität München, Munich, Germany

**Keywords:** metabolomics, resistance exercise, eccentric muscle contraction, hypoxia, metabolism

## Abstract

The aim of this study was to determine alterations of the metabolome in blood plasma in response to concentric-eccentric leg exercise performed at a simulated altitude of 3,500 m. To do so, we recruited 11 well-trained subjects and performed an untargeted metabolomics analysis of plasma samples obtained before, 20 min after as well as on day 8 after five sets of maximal, concentric-eccentric leg exercises that lasted 90 s each. We identified and annotated 115 metabolites through untargeted liquid chromatography-mass spectrometry metabolomics and used them to further calculate 20 sum/ratio of metabolites. A principal component analysis (PCA) revealed differences in-between the overall metabolome at rest and immediately after exercise. Interestingly, some systematic changes of relative metabolite concentrations still persisted on day 8 after exercise. The first two components of the PCA explained 34% of the relative concentrations of all identified metabolites analyzed together. A volcano plot indicates that 35 metabolites and two metabolite ratios were significantly changed directly after exercise, such as metabolites related to carbohydrate and TCA metabolism. Moreover, we observed alterations in the relative concentrations of amino acids (e.g., decreases of valine, leucine and increases in alanine) and purines (e.g., increases in hypoxanthine, xanthine and uric acid). In summary, high intensity concentric-eccentric exercise performed at simulated altitude systematically changed the blood metabolome in trained athletes directly after exercise and some relative metabolite concentrations were still changed on day 8. The importance of that persisting metabolic alterations on exercise performance should be studied further.

## Introduction

High altitude is associated with a reduced barometric pressure (P_B_) and a reduced inspiratory, partial pressure of oxygen (PIO_2_). At sea level, P_B_ and PIO_2_ are 760 and 149 mmHg, respectively, and these decline to 537 and 103 mmHg at 3,000 m, respectively (West, 2012). During exercise at high altitude, the altitude-associated drop of the PIO_2_ is accompanied by an increased, mitochondrial O_2_ uptake due to exercise. The body responds to the decreased oxygen availability during exercise under hypoxic conditions by increasing ventilation and cardiac output when compared to exercise at sea level (West, 2012). Also, if the rate of oxidative ATP resynthesis is reduced at a given workload, then anaerobic synthesis increases to compensate for impaired oxidative phosphorylation.

Metabolites are all small molecules of <1,500 Da present in a biological sample and are impacted by genetic background and environmental exposure, therefore metabolomics, that aims to characterize both the composition and amount of metabolites in a biological sample, can provide the ultimate molecular description of a phenotype (Nicholson, 2006). Traditionally, metabolites such as lactate were measured one by one using enzymatic reactions coupled to the detection of light absorption or fluorescence (Passonneau and Lowry, 2003). However, nuclear magnetic resonance and especially mass spectrometry (MS)-based metabolomics methods have allowed the unbiased detection of nearly 1,000 metabolites in microlitres of blood or other biofluids (Johnson et al., 2016). Given that exercise capacity is limited by the capacity and function of the Lohmann reaction (i.e., formation of ATP and creatine from ADP and phosphocreatine by creatine kinase), glycolysis and oxidative phosphorylation from fats and carbohydrates (Hargreaves and Spriet, 2020), metabolomics has been used to characterize the overall metabolic response to exercise (Contrepois et al., 2020; Morville et al., 2020; Schranner et al., 2020; Schranner et al., 2021). Metabolomics is also a suitable method to characterize the metabolic response to hypoxia alone or in combination with exercise (Davison et al., 2018).

Most metabolomics studies have investigated the blood metabolome response to endurance or resistance exercise. However, there are other types of exercise that are sometimes combined with hypoxia, e.g., to enhance adaptive processes which beneficially affect metabolism or the hematopoietic adaptations to hypoxia. The aim of this study was therefore to measure the metabolome in blood plasma before and after high intensity, concentric-eccentric exercise performed at a simulated high altitude where the oxygen content was lowered by ≈6% (fraction of inspired oxygen, FiO_2_ 0.146) when compared to normal air.

## Materials and Methods

### Subjects

Eleven male trained alpine ski athletes (age 30.4 ± 6.0 years, body height 1.81 ± 0.05 m, weight 76.4 ± 8.7 kg, HR_max_ 190 ± 8 bpm, VO_2max_ 53.9 ± 5.9 ml min^−1^ [mean ± SD]) volunteered to participate in this study. In the course of the medical examination in the days prior to the day of the intervention, a graded symptom-limited exercise test was performed on a bicycle ergometer (Lode B.V., Groningen, Netherlands) including gas analyses (Care Fusion, Vyntus CPX, Hoechberg, Germany). Participants were asked to refrain from exposure to altitude >2,500 m 2 weeks prior to and during the study as well as from consuming any antioxidant supplements throughout the entire study phase. The subjects were informed in writing and verbally about the study goals, procedures and potential risks. The study was carried out in accordance with the ethical standards laid down in the 1975 declaration of Helsinki and was approved by the local ethics committee.

### Experimental Design

**FIGURE 1:**
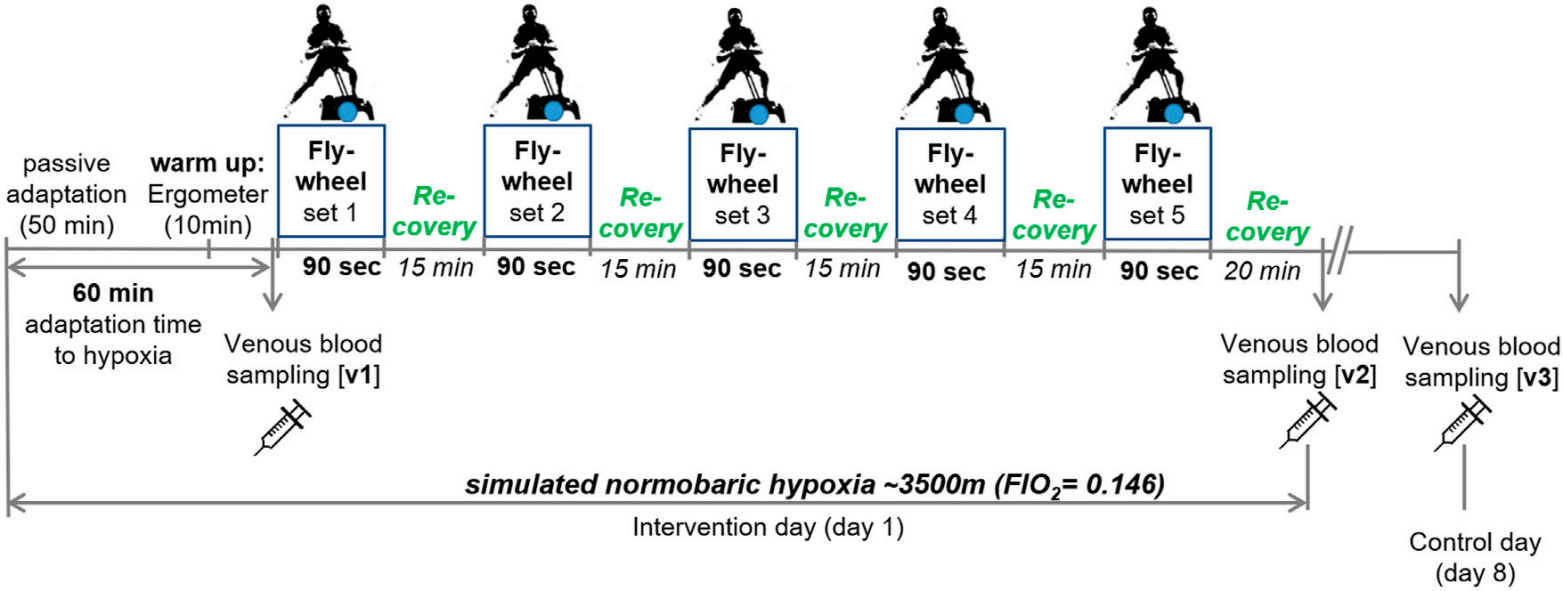
Outline of the study design. v1-3, venous blood sampling.

Eccentric leg exercise tests were performed on a flywheel device in an air-conditioned (21°C) normobaric hypoxic chamber (Low Oxygen, Berlin, Germany). Each subject was familiarized to the flywheel device 2 weeks before the beginning of the study experiments under normoxic conditions. After entering the hypoxic chamber, subjects performed a 45-min passive adaptation phase to hypoxia (FiO_2_ 0.146; ∼3,500 m), followed by a 10-min warm-up period on a cycle ergometer with an intensity determined at 1 W/kg. After a total of 60-min of adaptation to hypoxia (including warm up), venous blood (v1) was collected before starting with the first out of five flywheel sets, each lasting 90-s. Flywheel sets were separated by 15 min of passive recovery. Following the last flywheel set, subjects remained seated for 20 min in the hypoxic chamber. Venous blood samples were taken again immediately after the 20-min resting period (v3) and on day 8 following the intervention (control day; v3). Performance was evaluated for each flywheel set. An outline of the study design is shown in Figure 1.

### Flywheel Exercise

Subjects performed 90 s-lasting, high-intensity concentric-eccentric leg exercise sets (i.e., concentric-eccentric bilateral side-squats) on a k-Box device with an integrated flywheel (k-Box4 Pro, Exxentric AB, Sweden), as previously described in detail (Dünnwald et al., 2021). In short, the flywheel is connected to a rotating shaft with a band. On the other end, the band is fixed to the body of the exercising subject. Therefore, during the concentric phase of a side-squat, the flywheel accelerates by pulling out the band and rewinds on the shaft again, after a maximum length is reached. To slow down the kinetic energy of the flywheel, the muscles have to work eccentrically. In order to obtain a high power output during the eccentric phase, subjects were instructed to work at maximum velocity during the concentric phase. The kBox is equipped with a built-in kMeter that measures the rotation of the flywheel to obtain power output data (average power in one 90-s set; PO). For analysis, data were transferred via wireless transmitter to a mobile device and exported to excel.

### Venous Blood Sampling

We collected venous blood from the antecubital vein in the forearm (one EDTA sample; 5 ml) on the day if the intervention (v1, v2) and on the control day (v3). V1 and v3 were obtained at the same time of the day which was ∼2.5–3 h after a light breakfast. Blood was collected in EDTA tubes and inverted five times after blood drawing, blood EDTA samples were centrifuged at 2,500 rpm for 10 min at room temperature and the plasma obtained was stored at −80°C until the analysis. We then used the blood samples to perform an untargeted metabolomics analysis. This involved the separation of the metabolites by liquid chromatography followed by detection and quantification with mass spectroscopy. A control day was incorporated, as especially unfamiliar maximal eccentric exercises can lead to muscle damage, inflammation and extended recovery. Full recovery from moderate muscle damage was suggested to take up to 7 days and severe muscle damage to exceed 1 week (Paulsen et al., 2012).

### Untargeted Metabolomics

The plasma polar metabolome was analyzed using an LC/MS 6546 platform which includes an Agilent 1290 II liquid chromatography system (Agilent Technologies, Palo Alto, CA, United States) coupled to a time-of flight mass spectrometer (Agilent Technologies, Palo Alto, CA, United States). Chromatographic separation was achieved using hydrophilic interaction liquid chromatography (HILIC) by a Acquity amide column (100 × 2.1 mm, 1.7 μm) (Waters, Milford, MA, United States) (Volani et al., 2018). All samples were analyzed three times: once in positive ionization mode and twice in negative ionization mode using acidic and basic chromatographic conditions.

In the positive and negative acidic conditions, Mobile Phase A was 100% of 10 mM ammonium formate and B was 10 mM of acetonitrile:ammonium formate (95:5 v:v), both containing 0.1% formic acid. The following elution gradient was used: 0 min 98%, 1 min 98% B, 12 min 60% B, 13 min 50% B, 14 min 5% B, 15 min 5% B, 15.5 min 98% B, and 20 min 98% B.

In negative mode basic conditions, Mobile Phase A was 100% ammonium acetate 10 mM and B was acetonitrile: 10 mM ammonium acetate (95:5 v:v), both containing 0.1% acetic acid. The following elution gradient was used: 0 min 98%, 0.5 min 98% B, 6 min 30% B, 7.5 min 98% B, 12 min 98% B, and 15 min 5% B.

In all conditions, the flow rate was 0.25 ml/min, the column temperature was 40°C.

In ESI positive mode, the mass spectrometer operated at a resolving power of 40,000 over a full scan range of m/z 50–1,600 at a scan rate of 2 spectra/s with the following settings: gas flow 12 L/min; gas temperature 225°C; nebulizer 35 psi; sheath gas temperature 350°C; sheath gas flow 12 L/min; capillary voltage 3,500 V and fragmentator at 150 V.

In ESI negative mode, the mass spectrometer operated at a resolving power of 40,000 over a full scan range of m/z 50–1,600 at a scan rate of 2 spectra/s with the following settings: gas flow 6 L/min; gas temperature 225°C; nebulizer 40 psi; sheath gas temperature 225°C; sheath gas flow 10 L/min; capillary voltage 3,000 V and fragmentator at 150 V.

Purine was used as reference mass both in positive (m/z = 121.0509) and negative (m/z = 119.0360) experiments and continuously infused at flow rate of 0.08 ml/min.

Cold acetonitrile (150 μl) was added to 50 μl of plasma, and centrifuged at 15,000 g and 2 μl of supernatant were injected in the LC-MS system.

Pooled quality control (QC) samples were prepared by pooling together 10 μl of each sample. Ten injections of pooled QC were used to equilibrate the system at the beginning of each experiment and were also analyzed during the analysis (a QC sample was analyzed each 15 samples) to evaluate the stability of the LC-MS system.

Features with a relative standard deviation (RSD%) higher than 30 in pooled QC were removed and not considered for further analysis. At the end of the analysis, pooled QC samples were analyzed four times in Data-dependent acquisition (DDA) mode to acquire fragmentation spectra.

Data acquisition and analysis was done using the Agilent MassHunter software and Mass Profiler Professional. Annotated features were also integrated using Agilent MassHunter Quantitative software.

115 polar metabolites were annotated based on accurate mass, MS/MS, isotopic pattern and retention times against our in-house database and/or online databases, including HMDB (Wishart et al., 2018) and METLIN (Guijas et al., 2018).

### Statistics and Data Analysis

Metabolomics data analysis was performed by univariate and multivariate statistical analysis using MetaboAnalyst (Pang et al., 2021). Data was first normalized by the sum of the features and then log transformed and scaled by unit variance scaling method before applying multivariate analysis. Volcano Plots were obtained using a fold change threshold of 2 and a *p* value threshold of 0.05.

Performance data are presented as means ± standard deviation. A Kolmogorov-Smirnov test was applied to test for normal distribution of data. A repeated measures ANOVA was used to evaluate changes over time in PO of the flywheel exercise and for changes in mean heart rate. A *p*-value ≤ 0.05 was considered as statistically significant. All analyses were performed using the IBM SPSS statistical software package for Windows (version 24.0; IBM Corporation, Armonk, NY, United States).

## Results

### Exercise Performance

For this study, subjects performed five sets of eccentric exercise breathing air that contained 14.6% O_2_ at a normal barometric pressure. During these five sets, mean power output increased from 138 ± 29 W in the first set to 143 ± 32 W, 144 ± 29 W, 154 ± 31 W and 164 ± 33 W in sets two to five, respectively (*p* < 0.001; ηp^2^ = 0.44). Similarly, mean heart rate increased from 133 ± 14 bpm during set 1 to 137 ± 16 bpm (*p* = 0.65), 141 ± 17 bpm (*p* = 0.02), 142 ± 18 bpm (*p* = 0.04) and 144 ± 19 bpm (*p* = 0.12) during sets two to five, respectively (*p* = 0.027; ηp^2^ = 0.31).

### Do Relative Metabolite Concentrations Change in Response to Repeated Sets of Strength-Endurance Exercise Performed in Hypoxia?

To find out whether combined strength-endurance exercise under hypoxia alters the relative concentrations of blood metabolites when compared to resting conditions, we performed an untargeted metabolomic analysis of the plasma obtained from blood samples taken before exercise, immediately after the five sets of exercise as well as on day 8 after the exercise in hypoxia. Through untargeted metabolomics we obtained the profile of 135 metabolites and sum/ratios which are listed in Supplementary Table S1. As first step, for data visualization and to identify whole metabolome differences in the samples taken at rest, after five sets of exercise and on day 8 after exercise, we performed a principal component analysis (PCA).

**FIGURE 2:**
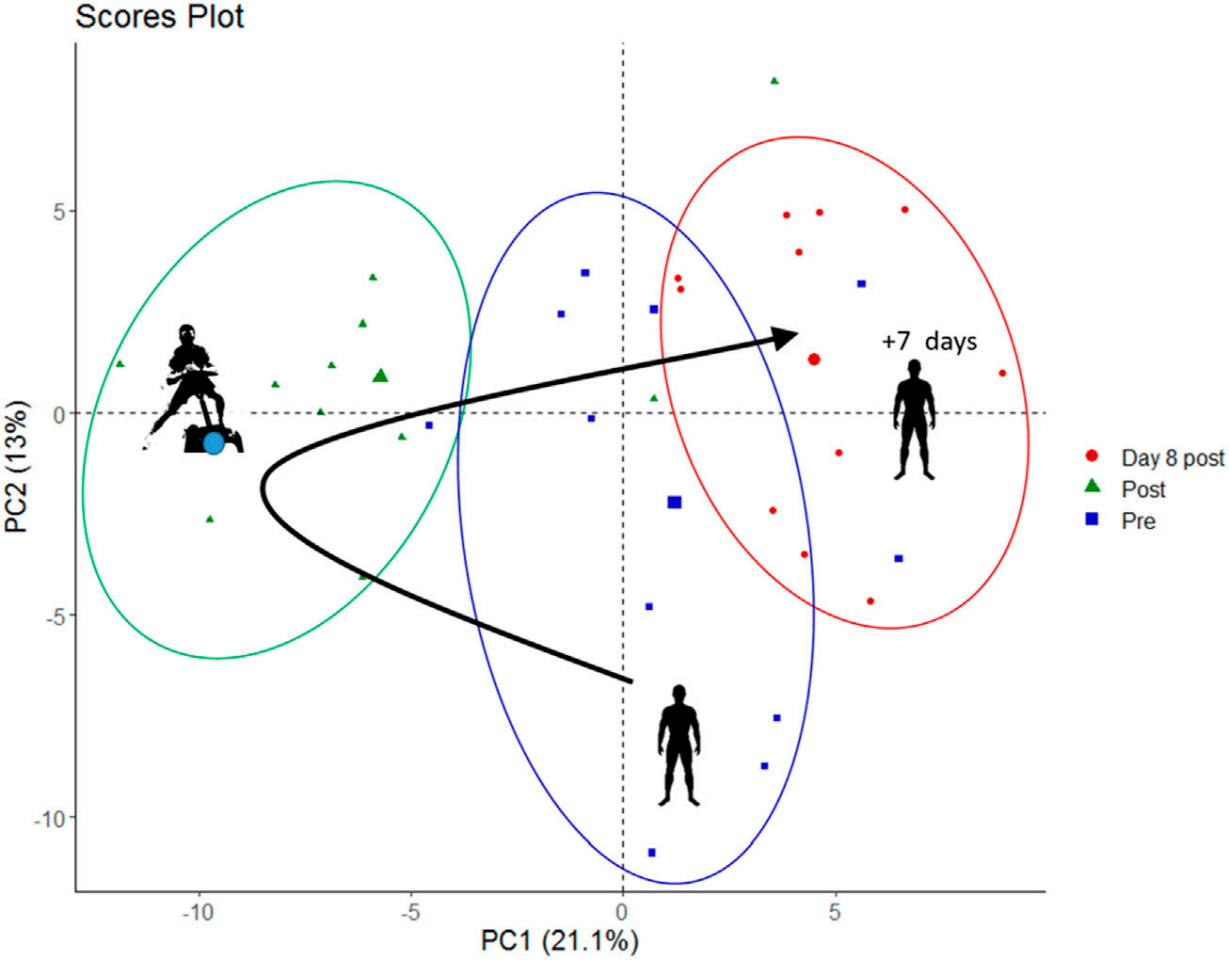
PCA score plot showing metabolite data from pre-exercise, immediately post-exercise and on day 8 post exercise (marked as blue squares, green triangles, and red circles, respectively).

The PCA plot (Figure 2) shows that the overall metabolome (i.e., the relative concentrations of all detected metabolites analyzed together) differed systematically in-between rest and after five sets of exercise, which was expected. Surprisingly, the metabolome was also still different on day 8 after the exercise, suggesting long-lasting changes after a bout of eccentric exercise. The first principal component (PC1, *x*-axis) contributed 21% of the total variance and the second principal component (PC2, *y*-axis) contributed 13% to the variation of relative blood metabolite concentrations in these samples.

We detected metabolites that can be linked to several metabolic pathways, ranging from carbohydrate metabolism (i.e., we observed expected increases of lactate and pyruvate), TCA cycle intermediates (increases of fumarate, succinate, malate), lipid metabolism (e.g., increases of acetylcarnitine and 3-Hydroxybutyrate), amino acids (e.g., increases of alanine, glutamate, as well as decreases of phenylalanine and valine) to purine metabolism (e.g., increases of uric acid and hypoxanthine). All changes are summarized in the heat map in Supplementary Figure S1. To identify metabolic pathways that were most altered by exercise we conducted a metabolite set enrichment analysis (MSEA) which is illustrated in Supplementary Figures S2 and S3. Selected hematological variables are presented in Supplementary Figure S4.

### What Metabolites Changed Most After One Bout of Resistance-Endurance Exercise?

**FIGURE 3:**
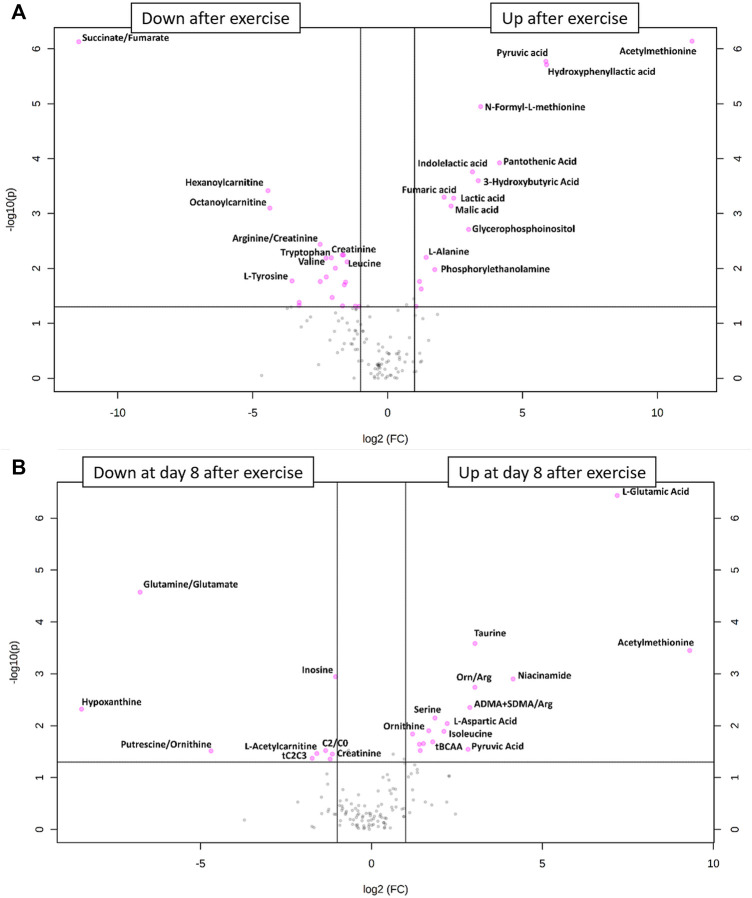
**(A)** Volcano plot of all metabolite alterations in the short-term **(A)** and on day 8 after exercise **(B)** showing the relationship between fold change (log2 fold change (FC); horizontal axis)) and significance (−log10 (*p* value); vertical axis), respectively. Fold changes describe alterations comparing post- to pre-exercise, using a cut-off point of *p* ≤ 0.05 and a FC ≥ 2. Metabolites shown as pink points indicate metabolites that were significantly down- or upregulated after **(A)** or on day 8 after **(B)** exercise, respectively.

Hypoxia and exercise are stimuli that are known to change the relative concentrations of blood metabolites such as lactate, pyruvate or glucose (Hargreaves and Spriet, 2020). To identify relative metabolite concentration changes from pre to post exercise, we drew a volcano plot (Figure 3A). This showed that 16 metabolites increased significantly whereas 19 metabolites and 2 metabolite ratios decreased after exercise as compared to their concentrations under resting conditions. Metabolites whose relative concentrations increased most were acethylmethionine, pyruvic acid and hydroxyphenyllacitc acid. The ratio succinate/fumarate decreased most prominently from pre to post exercise.

### Do Relative Metabolome Concentrations Return to Baseline on Day 8 After Exercise?

Especially eccentric exercise like the one performed by our subjects can cause exercise-induced muscle damage (Peake et al., 2017) which can take more than 7 days to regenerate (Paulsen et al., 2012). For this reason, we also drew blood samples 1 week after the bout of exercise to see whether there were long-term changes in metabolite relative concentrations. The results of this analysis are shown in the PCA plot (Figure 2) and in a volcano plot (Figure 3B) illustrating changes from baseline to day 8 after exercise. This analysis revealed systematic changes that are illustrated in Figure 3B. In detail, 14 metabolites and two metabolite ratios increased, whereas six metabolites and three metabolite ratios decreased compared to pre-exercise values. Acethylmethionine increased most on day 8 after exercise. Relative glutamic acid concentration increased and this change had the lowest *p*-value. The metabolite hypoxanthine and the metabolite ratio glutamine/glutamate decreased most.

**FIGURE 4:**
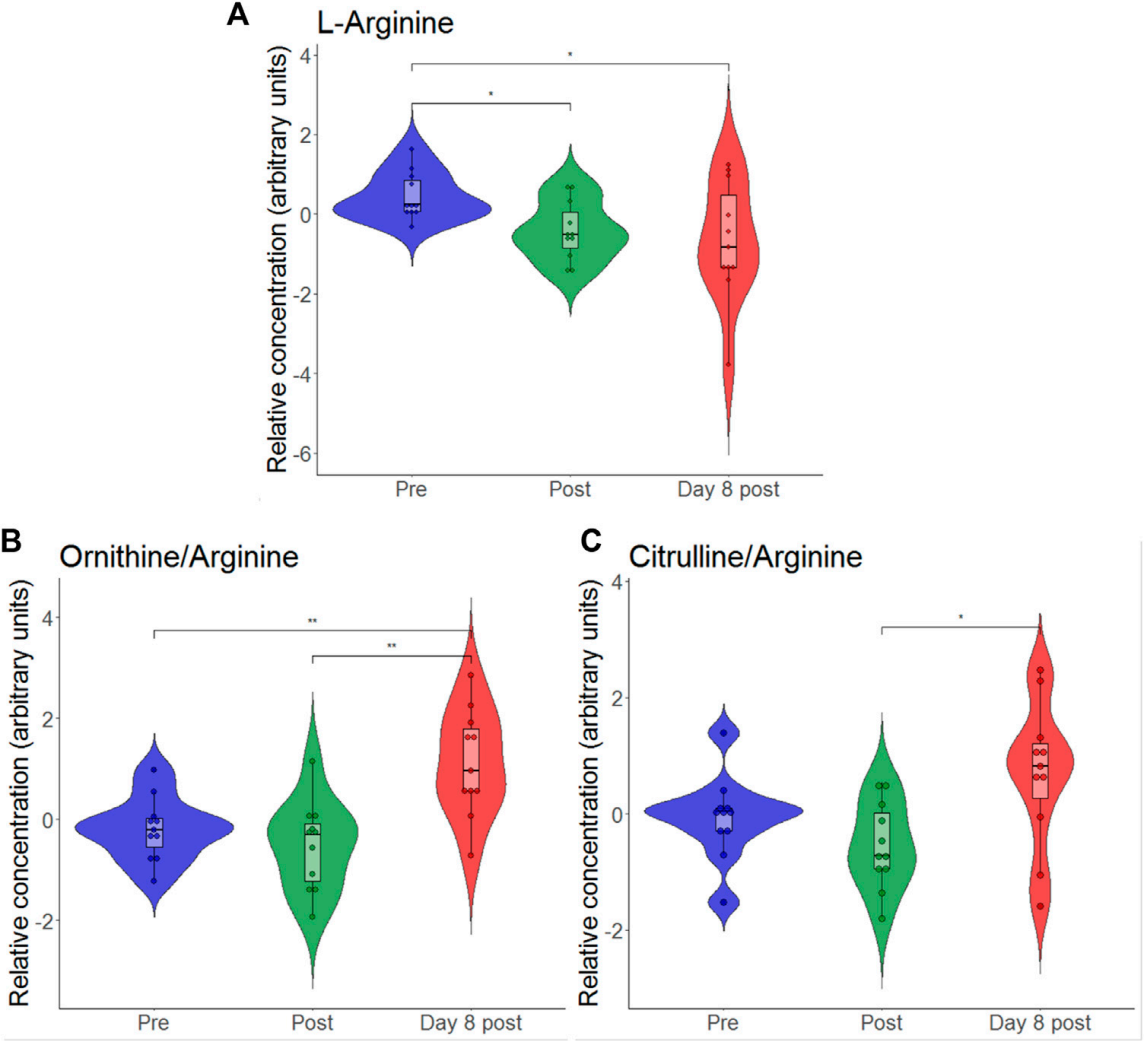
Violin plots with relative blood metabolite concentrations immediately after (post) and on day 8 after the eccentric exercise for L-arginine **(A)**, the ratio of ornithine-to-arginine **(B)** and the ratio of citrulline-to-arginine **(C)**. *p ≤ 0.05, *p ≤ 0.01.

According to the volcano plot, the ratio of asymmetric dimethylarginine (ADMA) and symmetric dimethylarginine (SDMA) to l-arginine increased on day 8 after exercise as compared to pre-exercise values. As the glucogenic amino acid l-arginine is involved in endothelial function via nitric oxide formation and as it has previously been shown to be reduced by intensive exercise (Nyborg et al., 2021), we individually plotted the relative concentrations of this metabolite. To find out how relative l-arginine concentrations change when exercise and hypoxia are combined, we created a violin plot (Figure 4). We found that l-arginine significantly decreased immediately after but also on day 8 after exercise in hypoxia.

**FIGURE 5:**
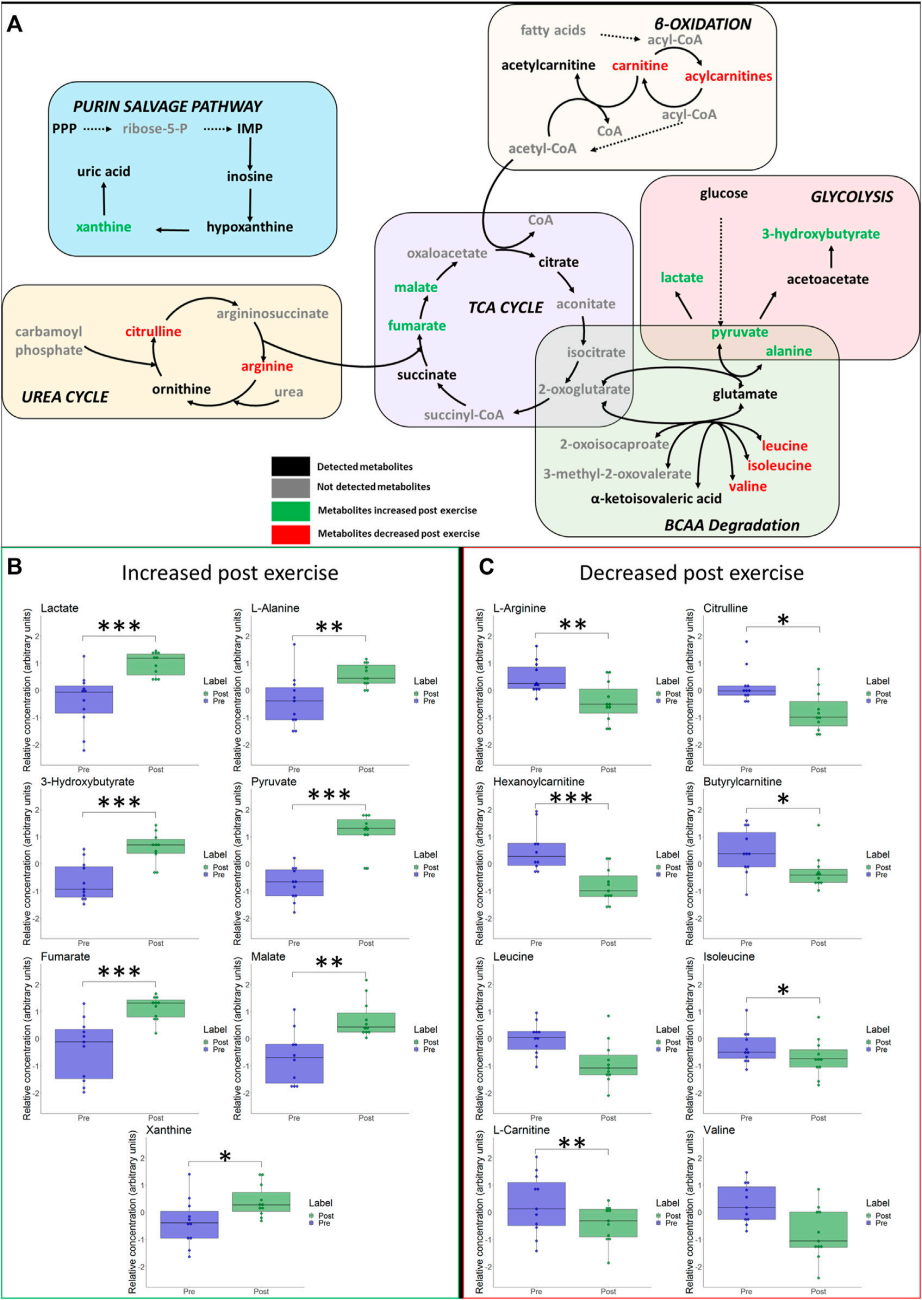
**(A)** Schematic overview of most important metabolic pathways. Changes of selected metabolites related to glycolysis, betta-oxidation, TCA-cycle, urea cycle and branched-chain amino acids (BCAA) immediately after hypoxic exercise are shown. Metabolites coloured as green represent metabolites that were upregulated immediately after exercise. Metabolites marked as red represent metabolites that were downregulated immediately after exercise. Metabolites marked as black represent detected metabolites whereas grey indicates non-detected metabolites. *p ≤ 0.05, **p ≤ 0.01, ***p ≤ 0.001 for significant increases **(B)** or decreases **(C)** in associated metabolites post exercise.

**FIGURE 6:**
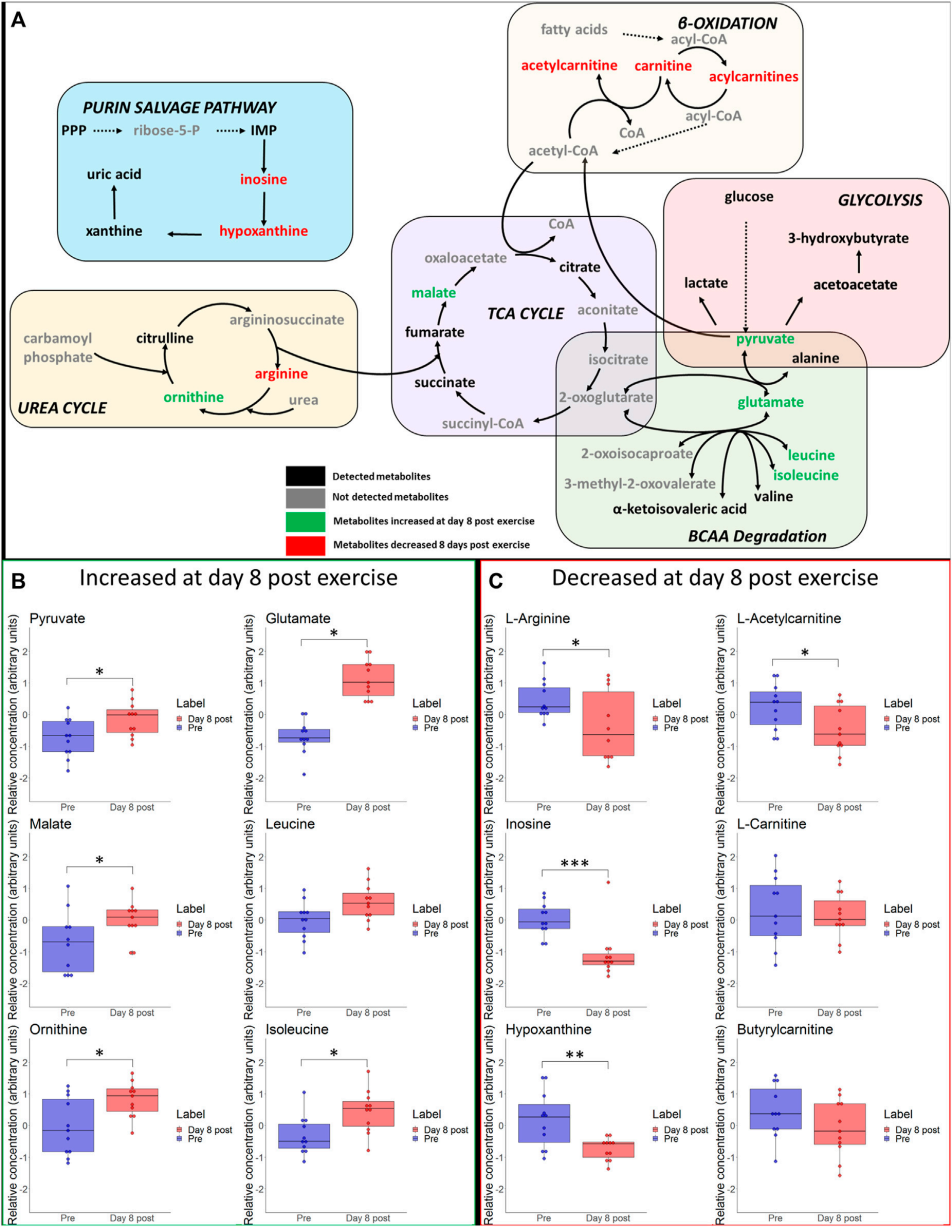
**(A)** Schematic overview of most important metabolic pathways. Changes of selected metabolites related to glycolysis, betta-oxidation, TCA-cycle, urea cycle and branched-chain amino acids (BCAA) at day 8 after hypoxic exercise are shown. Metabolites coloured as green and red represent metabolites that were upregulated and downregulated at day 8 after exercise, respectively. Metabolites marked as black represent detected metabolites whereas grey indicates non-detected metabolites. *p ≤ 0.05, **p ≤ 0.01, ***p ≤ 0.001 for significant increases **(B)** or decreases **(C)** in associated metabolites on day 8 post exercise.

Immediate- and long-term changes of selected metabolites of the main pathways are visualized in Figures 5 and 6, including box plots for significant changes.

### Response of Individual Metabolites to Hypoxia and Exercise

**FIGURE 7:**
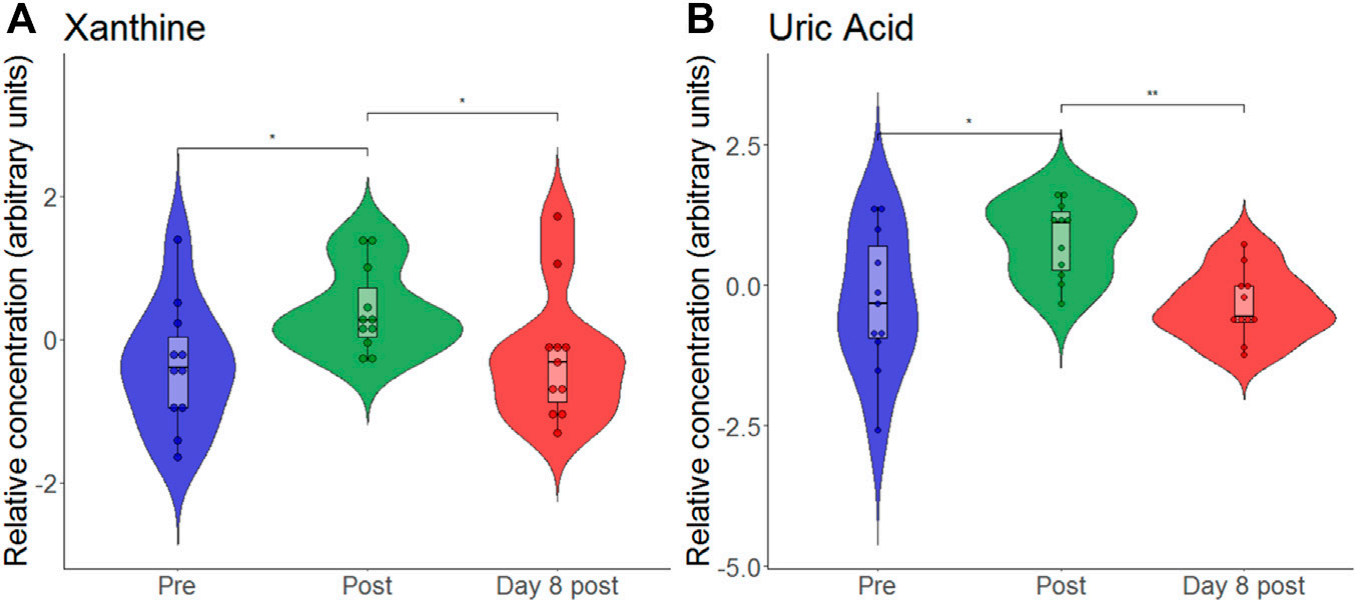
Violon plots showing relative blood metabolite concentrations at baseline before (pre), immediately after (post) and on day 8 after (day 8 post) the eccentric exercise for xanthine **(A)** and uric acid **(B)**. *p ≤ 0.05, **p ≤ 0.01.

At the end, we also analyzed the response of individual metabolites to hypoxia and exercise. We analyzed the purine metabolites uric acid, hypoxanthine and xanthine as they were previously discussed as biomarkers for hypoxia (Lou et al., 2014). To find out whether our data support this, we generated another violin plot of these metabolites (Figure 7). We found that both xanthine and uric acid significantly increased following eccentric exercise under hypoxia, but it is impossible to say whether the increase is due to hypoxia or exercise or both.

**FIGURE 8:**
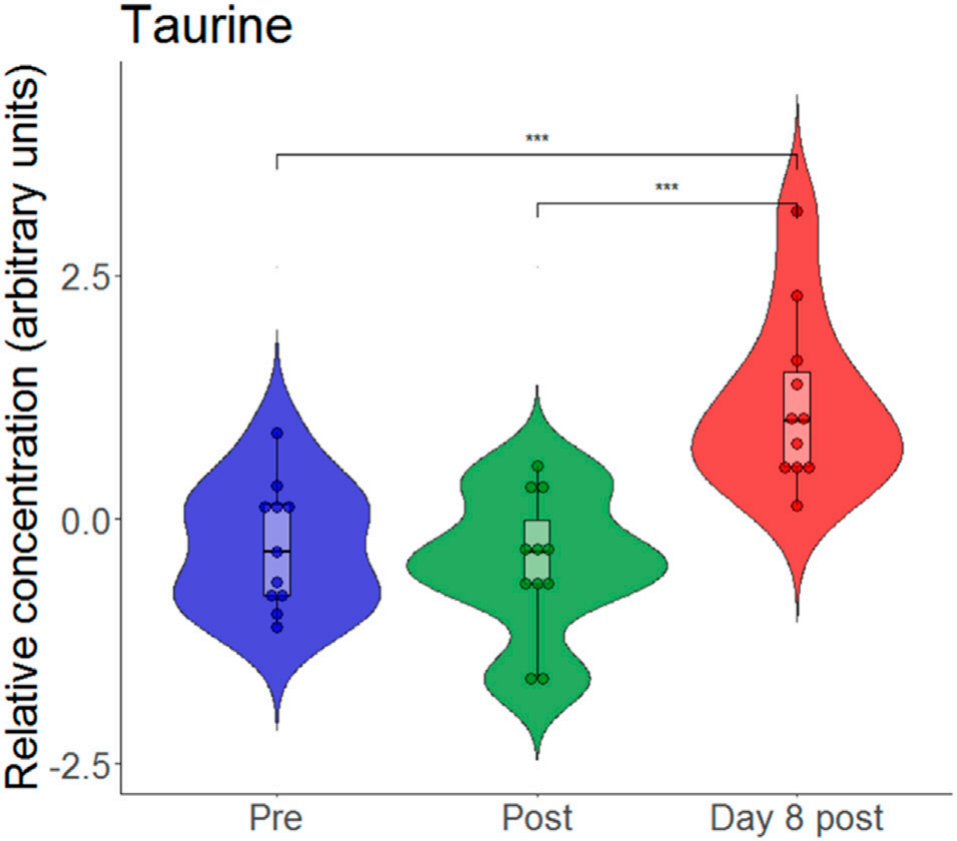
Violin plots with relative blood metabolite concentrations at baseline before (pre), immediately after (post) and on day 8 after (day 8 post) the eccentric exercise performed in hypoxia. ***p ≤ 0.001.

An animal model of hypoxia revealed that exposure to hypoxia without exercise can increase the relative concentration of taurine (Koundal et al., 2015). Again, we generated a violin plot (Figure 8) which revealed that taurine is unchanged directly after exercise but is increased on day 8.

## Discussion

The main finding of this study was that a bout of high intensity concentric-eccentric exercise at a simulated altitude of 3,500 m alters the blood metabolome in trained athletes. Surprisingly, some relative metabolite concentrations were still systematically changed on day 8 after exercise.

### The Blood Metabolome Is Acutely Changed by Hypoxic Exercise

Given that exercise (Schranner et al., 2020) and hypoxia (Lou et al., 2014) both affect relative metabolite concentrations, we expected extensive changes after a bout of hypoxic exercise, which was indeed the case. Globally, the first two components of the PCA score plot explained 34% of the total variance of all identified blood metabolites. PCA was able to discriminate between scores according to samples obtained during the different measurement time-points (i.e., before and after exercise), indicating hypoxic exercise to have a relevant impact on the variation of the overall blood metabolome immediately after exercise.

According to the volcano plots, several relative blood metabolite concentrations changed in response to hypoxic exercise. We found metabolites of carbohydrate metabolism and the TCA cycle were upregulated directly after completion of the bout of exercise. Similar changes are typically observed within the first 30 min following high intensity exercise in normoxia (Schranner et al., 2020), reflecting high usage of energy substrates such as muscle glycogen and blood glucose (van Loon et al., 2001; Morville et al., 2020).

Our data are in line with previous studies that reported that strenuous exercise (Berton et al., 2017; Morville et al., 2020; Schranner et al., 2020) reduced the relative concentrations of the branched-chain amino acids (BCCA) valine and leucine. Generally, BCAA contribute little to energy supply during short-term exercise unless carbohydrate availability is low (Hargreaves and Spriet, 2020). Leucine is used by the liver to synthesize ketone bodies such as 3-hydroxybutyrate, whereas the glucogenic amino acid valine is degraded to pyruvate and then synthesized to alanine (Pechlivanis et al., 2013; Berton et al., 2017). Alanine is transported via blood to the liver and is transformed to glucose (Pechlivanis et al., 2013; Schranner et al., 2020). Therefore, the increased relative concentrations of 3-hydroxybutyrate and alanine observed in our study can be ascribed to indirect energy provision by BCAA degradation in the recovery phase after the exhaustive exercise (Berton et al., 2017; Belhaj et al., 2021). Similar changes of relative BCAA concentrations occurred after prolonged exposure to high altitude (72-h bus travel to 5,300 m), with BCAAs being an alternative source of energy supply under hypoxia (Liao et al., 2016).

Another metabolite whose relative concentration increased was hydroxyphenyllactate. Hydroxyphenyllactate is synthesized when tyrosine is degraded. Hydroxyphenyllactate is functionally important because it is a natural antioxidant (Beloborodova et al., 2012).

Although we used high-load resistance exercise, our findings are in agreement with several of the metabolic changes observed in recent cycling studies analyzing red blood cells or whole blood in response to prolonged high intensity exercise and graded exercise to exhaustion, respectively (e.g., including alterations in energy metabolism, TCA cycle, amino acid- and purine metabolism) (San-Millán et al., 2020; Nemkov et al., 2021). This may indicate that even under environmental hypoxia, similar metabolic pathways can be activated by these two different types of exercise (i.e., strength or endurance exercise), provided that exercise intensity is high.

### The Blood Metabolome Is Still Systematically Changed on Day 8 After Eccentric Exercise Under Hypoxia

Because skeletal muscle can need up to 3 weeks to regenerate after injury and because eccentric exercise can induce muscle injury and delayed onset muscle soreness (DOMS) (Paulsen et al., 2012), we also analyzed the blood metabolome on day 8 after exercise. Even at this late stage, we detected systematic changes of relative blood metabolite concentrations.

The score plot of the PCA indicates that the metabolic profile on day 8 is still different from that observed at baseline. There is little research evaluating metabolite relative concentration changes over a comparable period. However, several long-term alterations in urinary metabolites in response to exercise induced muscle damage were reported reaching maximum levels within 48 h after the exercise (e.g., increased lactate, citrate, glucose, histidine), with the relative concentrations of alanine, glycine and formate still being elevated until 72 h (Jang et al., 2018).

Here, we observed that hypoxic exercise resulted in significant reductions in relative l-arginine concentrations immediately after and on day 8 after exercise. According to the volcano plot analysis, these changes were accompanied by increased ratio of asymmetric dimethylarginine (ADMA) and symmetric dimethylarginine (SDMA) to l-arginine in the long-term. Similarly, when consideration is also given on the catabolic products of arginine (i.e., citrulline and ornithine), significant increase in the ratio of citrulline-to-arginine and a nonsignificant increase in the ratio ornithine-to-arginine appeared. Hypoxia and exercise may be independent factors to impact on arginine metabolism, as arginine was reported to be downregulated in the early phase of exposure to high altitude hypoxia (D'Alessandro et al., 2016) but also immediately after and the next day after prolonged strenuous exercise (i.e., ironman marathon) (Nyborg et al., 2021). Arginine is a known substrate for nitric oxide (NO) synthesis which regulates endothelial vasodilatory function (Madeo et al., 2018). Interestingly, systemic low-grade inflammation was related to reduced plasma arginine levels (van der Zwan et al., 2011), which is likewise being due to consumption of l-arginine by inducible nitric oxide synthase and Arginase-1, both being induced by inflammatory cytokines (Brigo et al., 2021). In addition to arginine, accounting for amino-acid ratios may be of interest regarding NO metabolism, especially endothelial function. Previous studies revealed arginine bioavailability ratios (i.e., arginine-to-ornithine ratio and global arginine bioavailability ratio (GABR; arginine divided by the sum of ornithine plus citrulline)) to be related to reduced endothelial function in patients with type 2 diabetes mellitus (Sourij et al., 2011; Kövamees et al., 2016), and increased plasma citrulline- to-arginine ratios to be associated with blood pressure abnormalities in children with early chronic kidney disease (Lin et al., 2013).

### Hypoxia-Associated Individual Metabolite Responses

Our findings of increased accumulation of nucleotide degradation products (i.e., hypoxanthine, xanthine, uric acid) in the short-term confirm prior observations from high intensity endurance or resistance exercises performed in normoxia (Berton et al., 2017; Schranner et al., 2020). Massive changes in xanthine and uric acid in our study can be ascribed to the xanthine oxidase mediated oxidization of hypoxanthine, a purine metabolite that was found to be associated with tissue hypoxia (Ihara et al., 2001). No such changes were observed following submaximal-intensity hypoxic exercise (FiO_2_: 0.16) (Davison et al., 2018). Authors suggested that this absence of change is attributed to the low intensity of the exercise that prevented ATP degradation (Davison et al., 2018). Contrastingly, passive acute exposure to two different altitudes (i.e., 3,000 m and 4,500 m) significantly increased the relative concentration of hypoxanthine, with xanthine and uric acid representing a dose dependency with more pronounced changes during passive exposure at higher altitude (Lou et al., 2014). These findings of an isolated effect of the hypoxic stimulus were confirmed by another study that revealed increases in hypoxanthine and uric acid together with a significant upregulation of xanthine oxidase after a 4-day stay at high altitude (5,300 m), (Liao et al., 2016).

On day 8 after exercise, levels of the biogenic amine taurine were elevated. Higher levels of taurine were observed following an 8-week high intensity sprint interval training (Pechlivanis et al., 2013). Moreover, hypoxic exposure alone can increase the relative concentration of taurine (Koundal et al., 2015; Sibomana et al., 2021). According to its anti-inflammatory and anti-oxidative properties, taurine was considered as a cytoprotective molecule (Baliou et al., 2021; Kurtz et al., 2021). As such, taurine supplementation was analysed in several studies that investigated its effect on athletic performance (Kurtz et al., 2021) as well as on oxidative stress-related disorders (Baliou et al., 2021). In experimental hypoxia, the taurine pathway was one of the most altered components of metabolism (Koundal et al., 2015). Interestingly, subjects that developed symptoms of acute mountain sickness (AMS) exhibited lower taurine excretion levels during both conditions, at sea level and after the first day at high altitude when compared to non-AMS subjects (Sibomana et al., 2021).

The underlying mechanism for these long-term metabolic adaptations are currently unclear but others also found persistent relative concentration changes long after a bout of exercise. For example, recent studies reported relative concentration changes of nitrogen-, arginine- and sulphur metabolism together with improved performance or a preserved hypoxic adenosine response. This might hint at a “metabolic memory” upon reascending 1 week after a prolonged stay at altitude (D'Alessandro et al., 2016; Song et al., 2017). Future studies should investigate whether stimuli such as exercise or hypoxia can form a “metabolic memory”.

This study has limitations. First, post-exercise venous blood sampling was performed after a 20-min resting phase in hypoxia. Thus, the magnitude of changes may differ when drawing samples directly after cessation of the exercise. Another limiting factor could be that baseline blood collection occurred after a 60-min adaptation phase to hypoxia. This should be kept in mind when interpreting changes in the relative blood metabolite concentrations observed on day 8 after the hypoxic exercise. Further, subjects were exposed to normobaric hypoxia. Therefore, some changes in the metabolome may differ when subjects are exposed to hypobaric hypoxia (e.g., due to the possibility for higher oxidative stress at hypobaric hypoxia). Finally, exercise was performed in trained skiers and therefore changes in the metabolomic profile may vary in athletes that are less used to exhaustive exercises with accompanied high falls in oxygen availability. In addition, we did not evaluate other blood parameters which may affect mitochondrial function, such as iron or glucose levels, thereby impacting on metabolic signatures or the relative ratio of aerobic versus anaerobic glycolytic activity (Volani et al., 2018). Another limitation is that we have only analyzed blood plasma and no blood cells. Other studies have analyzed the effect of exercise on whole blood (San-Millán et al., 2020) or the effect of high-altitude exposure on red blood cells or muscle (D'Alessandro et al., 2016; Chicco et al., 2018). Lastly, we performed only limited longitudinal sampling.

Beside these limitations, we were able to show that metabolomics can help to identify the myriad of blood metaboliteresponses to exercise performed in hypoxia. Metabolomics allows to depict hundreds of metabolites in one single sample and therefore, compared to individual measurement of metabolites (e.g., lactate), a comprehensive view on exercise induced changes in metabolite concentrations can be given. In the future, if biomarkers can be detected by means of metabolomics (e.g., for metabolic function, training status trainability etc.), development of standard curves for selected metabolites is imaginable (e.g., a combination of 10 metabolites) that enable to obtain real metabolite concentrations. In the area of exercise physiology, this would increase the practical relevance specifically for sports physiologists, who are used to do concentration curves.

In summary, we observed systematic alterations of relative blood metabolite concentrations in trained athletes in response to a single session of high intensity eccentric exercise performed during a simulated altitude of 3,500 m. However, we are not able to discriminate if these substantial changes are mainly due to exercise, hypoxia, or both. Moreover, our findings indicate that some relative metabolite concentrations can still be altered on day 8 after exercise. There is a need for further research to evaluate potential additive effects or interactions of these two stressors (i.e., exercise and hypoxia), using various altitude levels and to evaluate the impact of these persisting metabolic alterations on exercise capacity and endurance.

## Data Availability

The raw data supporting the conclusions of this article will be made available by the authors, without undue reservation.
