# Supplementary material for: High Intensity Concentric-Eccentric Exercise Under Hypoxia Changes the Blood Metabolome of Trained Athletes
Source: Front Physiol. 2022 Jun 23;13:904618. doi: 10.3389/fphys.2022.904618 (PMC9260056; doi:10.3389/fphys.2022.904618)
Supplement: Supplementary file 2 [file DataSheet3.PDF]

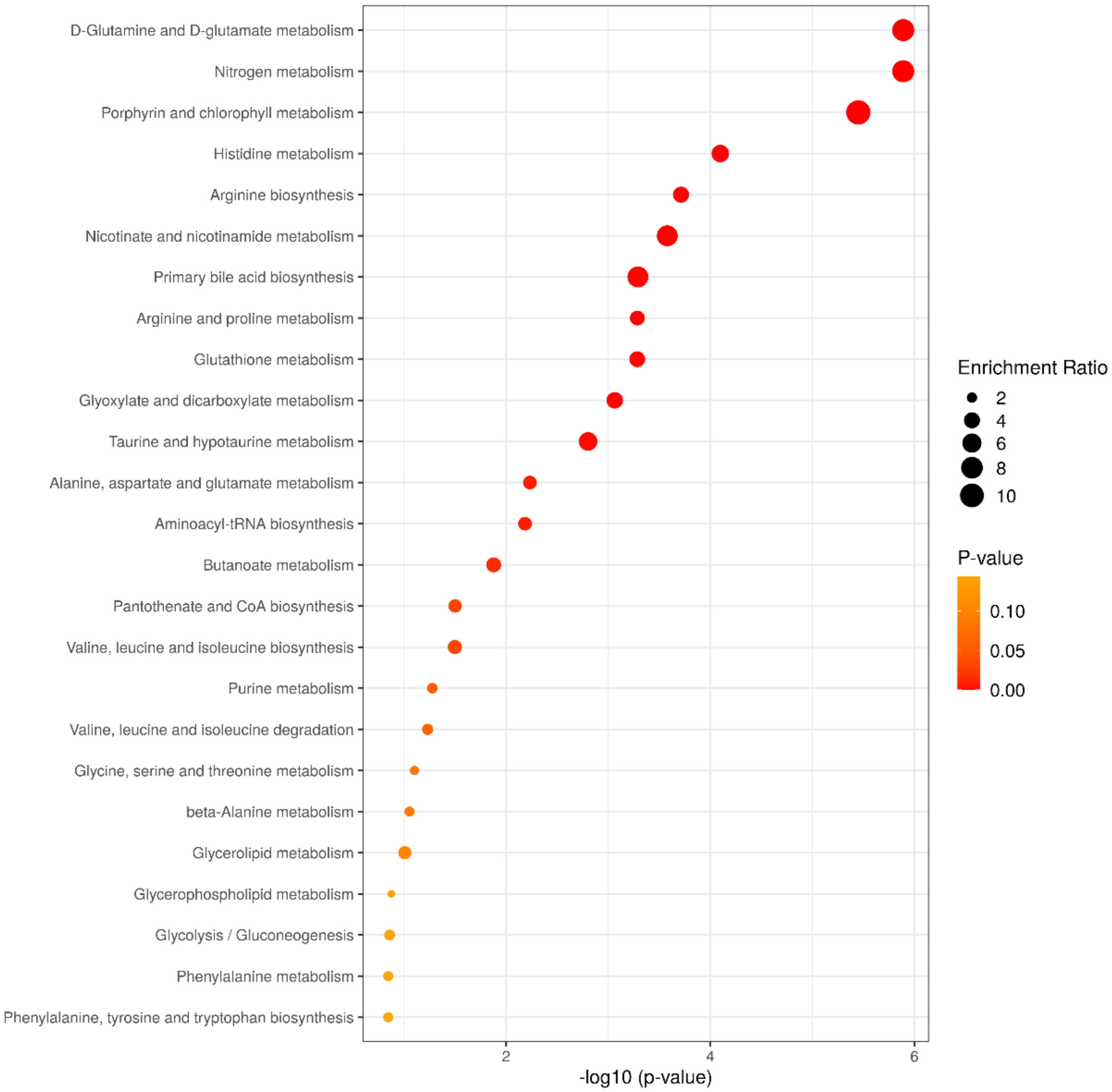

Supplementary Figure 3. Heat map of the top 25 significantly enriched metabolite sets from metabolite concentrations. Size of the dots per metabolite set displays the count of enriched metabolites in the pathway (pathway impact) and the color of the dots shows the pathway enrichment significance. Pathways modified on day 8 after hypoxic exercise.
