# Supplementary material for: High Intensity Concentric-Eccentric Exercise Under Hypoxia Changes the Blood Metabolome of Trained Athletes
Source: Front Physiol. 2022 Jun 23;13:904618. doi: 10.3389/fphys.2022.904618 (PMC9260056; doi:10.3389/fphys.2022.904618)
Supplement: Supplementary file 3 [file Table2.DOCX]

**Supplementary Table 2** Changes of hematological variables

|  | Pre | Post | Day 8 post |
| --- | --- | --- | --- |
| Erythrocytes [*10^12^/L] | 5.00±0.38 | 4.99±0.40 | 4.76±0.35* |
| Hemoglobin [g/L] | 149.91±7.67 | 149.64±7.79 | 142.73±6.87* |
| MCH [fmol] | 1.87±0.07 | 1.87±0.08 | 1.81±0.07 |
| Hematocrit [%] | 0.43±0.03 | 0.43±0.03 | 0.41±0.02* |
| Reticulocytes [‰] | 53.96±15.22 | 54.28±15.71 | 55.46±16.52 |
| Leucocytes [*10^9^/L] | 5.77±1.52 | 5.69±1.51 | 5.81±1.75 |

Data are mean ± standard deviation. A p-value ≤ 0.05 was considered as statistically significant; no significant changes in erythrocytes, hemoglobin, hematocrit, MCH, reticulocytes or leucocytes from pre (after 60 minutes adaptation to hypoxia) to immediately post exercise (p>0.05). *for significant changes from pre- exercise to day 8 after exercise (p≤0.05).
