# Supplementary material for: High Intensity Concentric-Eccentric Exercise Under Hypoxia Changes the Blood Metabolome of Trained Athletes
Source: Front Physiol. 2022 Jun 23;13:904618. doi: 10.3389/fphys.2022.904618 (PMC9260056; doi:10.3389/fphys.2022.904618)
Supplement: Supplementary file 4 [file DataSheet1.PDF]

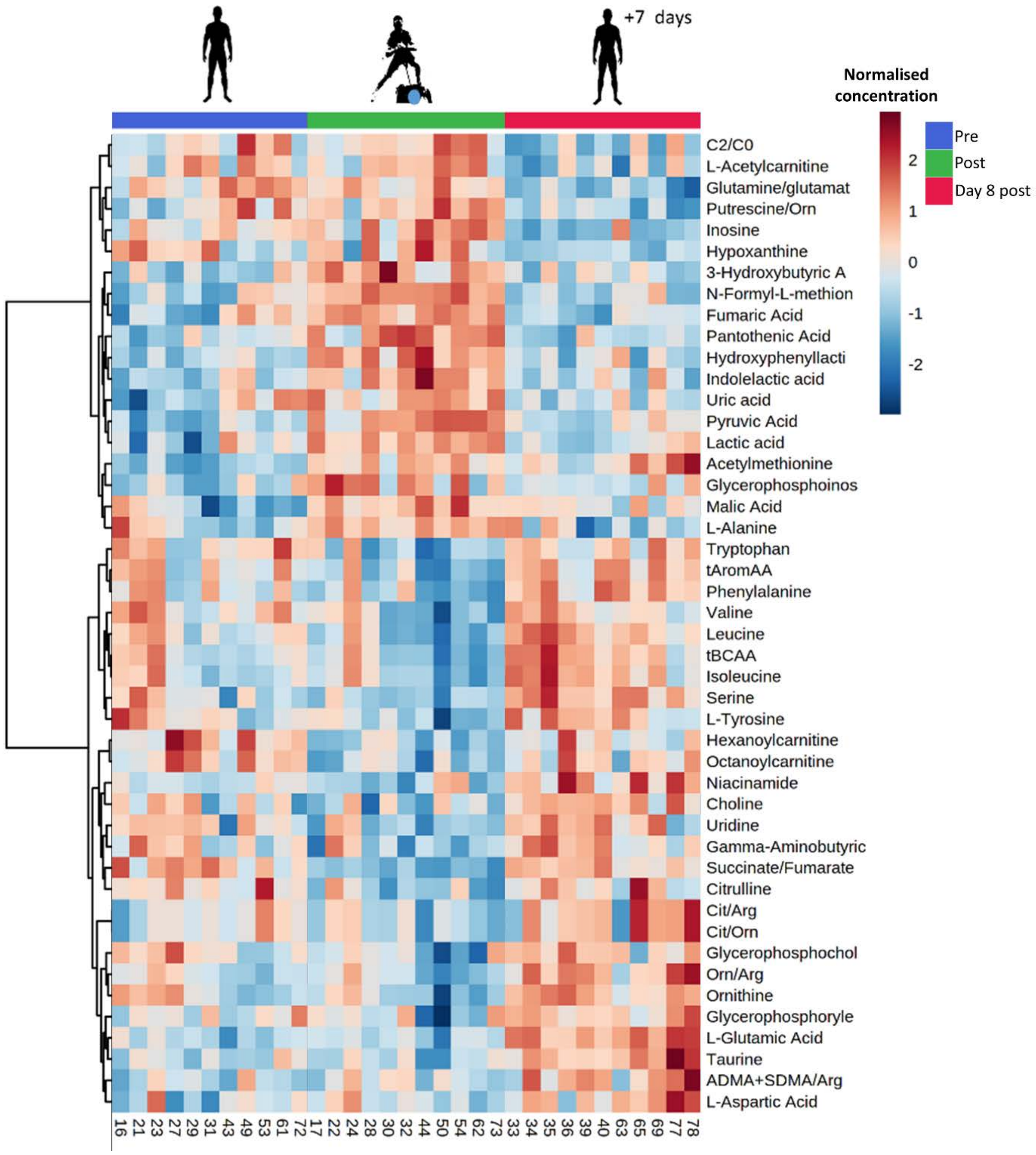

Supplementary Figure 1. Heat map with hierarchical clustering including differentially 46 regulated metabolites from blood samples collected at baseline (blue bar), post-exercise (green bar) and at day 8 post exercise (red bar) in 11 male subjects (y-axis). Color key illustrates metabolite expression values as normalized concentrations ranging from 2 (highest increase; marked as red) to –2 (highest decrease; marked as blue). Dendogram on the left indicates both, the similarity and the order that clusters were formed. Most similar cluster has the shortest branch.
